# Using Reputation Systems and Non-Deterministic Routing to Secure Wireless Sensor Networks

**DOI:** 10.3390/s90503958

**Published:** 2009-05-25

**Authors:** José M. Moya, Juan Carlos Vallejo, David Fraga, Álvaro Araujo, Daniel Villanueva, Juan-Mariano de Goyeneche

**Affiliations:** Dpto. Ingeniería Electrónica, Universidad Politécnica de Madrid, ETSI Telecomunicación, Av. Complutense, 30, 28040 Madrid, Spain

**Keywords:** security, reputation system, wireless sensor networks, routing protocol, sybil attack, countermeasure

## Abstract

Security in wireless sensor networks is difficult to achieve because of the resource limitations of the sensor nodes. We propose a trust-based decision framework for wireless sensor networks coupled with a non-deterministic routing protocol. Both provide a mechanism to effectively detect and confine common attacks, and, unlike previous approaches, allow bad reputation feedback to the network. This approach has been extensively simulated, obtaining good results, even for unrealistically complex attack scenarios.

## 1. Introduction

In essence, an intelligent environment is a distributed system that collects data from a wireless sensor network, processes this data, and enriches the environment with new meaning. These semantic enhancements can be used by other applications running on top of our system to make decisions.

Security concerns are key issues in ambient intelligence (AmI) since its earliest inception [1]. Many researchers clearly recognize the inherent challenge that an invisible, intuitive and pervasive system of networked computers holds for current social norms and values concerning privacy and surveillance. In fact, the increasing attack rate has become the bottleneck of adopting next-generation services and applications. A study from the Computer Security Institute reveals that a random sample of 223 organizations had lost hundreds of millions of dollars in 2002 due to security attacks [2].

For example, Brumley and Boneh [3] developed a timing attack for the OpenSSL implementation of RSA in a real TCP/IP network. This low-cost attack exploits some asymmetries introduced by two optimizations used in the OpenSSL implementation. Even in OpenSSL, that is considered to be quite reliable and secure, and it is used in many servers around the world, it is possible to find asymmetries that reveal some data of the cryptographic keys. And these asymmetries can be used to implement a real attack. Using OpenSSL or something equivalent for sensor communications would be impractical in most cases, and therefore the security threats become much worse as many more attack opportunities arise.

Three factors contribute to make security in wireless sensor networks a very difficult problem: 1) many nodes in the network have very limited resources; 2) pervasiveness implies that some nodes will be in non-controlled areas and are accessible to potential intruders; 3) all these computers are globally interconnected, allowing attacks to be propagated step by step from the more resource-constrained devices to the more secure servers with lots of private data.

Usually, security issues are addressed, in a similar way to services in a network of general-purpose computers, by adding an authentication system and encrypted communications. First, the resource limitations make the embedded computers especially vulnerable to common attacks.

In previous work [4], we demonstrated that current ciphers and countermeasures often imply a need for more resources (more computation requirements, more power consumption, specific integrated circuits with careful physical design, etc.), but usually this is not affordable for this kind of applications. But even if we impose strong requirements for any individual node to be connected to our network, it is virtually impossible to update hardware and software whenever a security flaw is found. The need to consider security as a new dimension during the whole design process of embedded systems has already been stressed [5, 6], and there have been some initial efforts towards design methodologies to support security [7–9], but to the best of our knowledge no attempt has been made to exploit the special characteristics of wireless sensor networks.

Applications built on wireless sensor networks have to live with the fact that privacy and integrity cannot be preserved in every node of the network. This poses restrictions on the information a single node can manage, and also in the way the applications are designed and distributed in the network.

Of course, the inherent insecurity of embedded systems should not lead us to not try hard to avoid compromises. We should guarantee that a massive attack can not be fast enough to avoid the detection and recovery measures to be effective. Therefore we should design the nodes as secure as the available resources allow.

In spite of the disadvantages of wireless sensor networks from the security point of view, they provide two advantages for fighting against attacks:

- Redundancy. A wireless sensor network usually has a high degree of spatial redundancy (many sensors that should provide coherent data), and temporal redundancy (habits, periodic behaviors, causal dependencies), and both can be used to detect and isolate faulty or compromised nodes in a very effective manner.
- Continuous adaptation. Wireless sensor networks are evolving continuously, there are continuous changes of functional requirements (data requests, service requests, user commands…), nodes appear and disappear continuously and therefore routing schemes change, low batteries force some functionality to be migrated to other nodes, etc.

In this article we propose a more secure approach to the design of applications built on a wireless sensor network by exploiting these two properties. In Section 2 we review some of the most relevant previous approaches. Section 3 describes our approach in detail. In Section 4 we review some relevant attacks, the countermeasures that have been proposed previously, the requirements that these threats impose to our design strategy and demonstrates how this approach can detect and confine them. In Section 5, some experimental data is shown and discussed. Finally, in Section 6, we draw some conclusions.

## 2. Related Work

Many research works have dealt with the problem of security in wireless sensor networks. Buttyán and Hubaux [10, 11] proposed an architecture to stimulate a correct routing behavior. In their solution, nodes receive a per-hop payment in every packet they forward. Nodes store this payment information in an internal counter. As elements get benefits from routing, they understand cooperation as a benefit not only for the entire network, but also for individual nodes. This approach, however, maintains this cooperation information locally, only shared by nodes that interact directly. In our approach, on the contrary, reputation information is transmitted all over the network, so all nodes are warned about misbehaving nodes.

Marti *et al.* [12] proposed mitigating routing misbehavior by detecting non-forwarding nodes and rating every path so those nodes are avoided when the routes are recalculated. The resulting behavior is that non-routing nodes are not included in routing paths (as they are not going to cooperate) but they still can ask others to forward their messages. According to Dawkins [13], this scheme detects the misbehavior but it does not isolate it. In our system, bad-behaving or attacker nodes are isolated by rating their reputation as low.

Many approaches to secure wireless sensor networks use encryption keys, or need user authentication and/or authorization. Stajano and Anderson [14] accept encryption keys from the first device that sends such a key. Smith, Murthy and Garcia-Luna-Aceves [15] studied vulnerabilities of distance vector protocols and proposed countermeasures for these possible attacks. Their solution includes sequence numbers and digital signatures so senders can be identified and messages from attacking or misbehaving nodes are not routed. We opt for detecting bad behavior and dropping the reputation for these nodes. We assume misbehavior and attacks will happen and the system is responsible for isolating them.

The CONFIDANT solution, proposed by Buchegger and Le Boudec [16] is a good effort that solves most of the problems mentioned in this section. CONFIDANT is a protocol that sits over the chosen routing protocol and makes misbehavior less attractive for the nodes than proper routing: nodes watch their neighbors for bad behavior, take into account this behavior in a local reputation system and eventually inform their trusted neighbors on misbehaving nodes. Our approach is in some aspects similar to CONFIDANT. For example, it has a reputation system too, but it is not local, but global.

Intuitively, a node *i* should give more weight to the direct observations made by it than the evidence obtained from other nodes. Furthermore, the evidence from different nodes should be weighted on the basis of their respective reputations. The beta reputation system [17] and recent implementations for Mica2 motes [18] base on these observations to provide resistance against bad-mouthing attacks. A different scheme is proposed in [19], based on the separation between action trust and recommendation trust.

Either because they hold encryption keys or for other reasons, many approaches demand that the nodes be tamper-proof secure [20]. But this need is more difficult to fulfill with every passing day [21–23]). CONFIDANT's authors reason out that their protocol does not need tamper-proof hardware because other nodes' reputation tables are not alterable by the attack. As far as we know, it is not very clear how they maintain the local trusted nodes list or how trust information is updated. It could be difficult to detect a sybil attack [24, 25] or the impersonation of a trusted node by first reading the trusted node list from a tampered node.

In an early design stage we decided not to depend on the hardware being tamper-proof. In fact, it is our assumption that it isn't *and* that the communications between nodes with limited resources are not secure. Our approach compensates those drawbacks by taking advantage of redundancy, temporal and spatial.

In general, most of the studied architectures provide security (by just preventing attacks or by simultaneously detecting attacks and providing countermeasures) in the routing protocol, at network level. Our security infrastructure is designed for intelligent environments, so it takes advantage of the environment and uses information from the application layer.

## 3. Our Approach

### 3.1. Overview

We focus on the development of secure applications in future wireless sensor networks, where many sensors provide data about observable magnitudes from the environment, and many actuators let the system act on the state of the environment.

Following the Ackoff taxonomy for the content of the human mind, we classify the content of the “ambient mind” into four categories:

1. Data: Symbols. It simply exists and has no significance beyond its existence (in and of itself).
2. Information: Data that is processed to be useful; provides answers to “who”, “what”, “where”, and “when” questions.
3. Knowledge: Application of data and information; answers “how” questions.
4. Intelligence (actually, this category comprises two from the Ackoff taxonomy: understanding and wisdom): Appreciation of “why”. It is the process by which new knowledge is synthesized from the previously held knowledge.

The main characteristic of an intelligent ambient is the semantic enrichment of environment based on the processing of data obtained from the environment using a sensor network. This “ambient mind” enhances the semantics of the environment by adding meaning to the objects. The objects are conscious of the “who”, “what”, “where”, “when”, “how”, and “why”.

Data is obtained by sensor nodes, but as they are not trusted, most of the remaining processing should be done in secure servers so that confidentiality attacks do not succeed (note that data has no meaning by itself). Data is sent to servers where it is processed to generate information, and then knowledge, and then understanding, and then new meaning, which is returned back to the environment. Individual nodes may be insecure, but the system should always continue its function of semantic enhancement. Moreover, attacks of individual nodes should not affect the decisions based on data from the environment. These requirements are achieved by perusing redundancy to discard data from the compromised nodes, and by changing the network structure and behavior at a speed that is fast enough to prevent a chained attack to spread.

**Figure 1.**
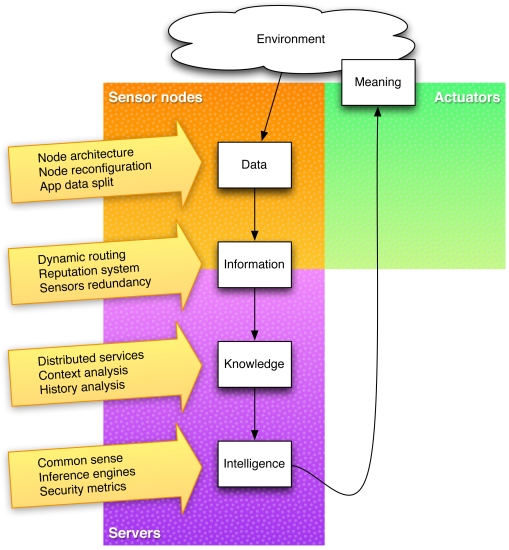
Overview of the data processing flow in an intelligent environment and the security measure we adopt.

Figure 1 shows the data flow in the environment. As confidentiality attacks become more dangerous as data is further processed, there should be little or no processing at all in the sensor nodes, which are more vulnerable.

## 3.2. Network Model

We consider the network composed of two kinds of nodes: wireless nodes and servers.

- Wireless nodes. They provide data to the network to enable decisions to be made. In our model, decisions are made primarily in secure servers, and therefore the main task of these wireless nodes is sending data to the servers. The more data is sent to the servers, the more redundancy can be used to discard bad data and to detect failures or intrusions. But also, the more data is sent, the more bandwidth is used and the more energy is consumed, so we have to reach a compromise. There are many wireless nodes in an intelligent ambient, so they have to be inexpensive, what usually means very limited resources, battery-powered, not maintained and hence insecure; an intruder may have physical access to them.
- Servers. They receive data from sensors and make decisions in order to reach the applications objectives. These decisions may imply to act in the environment and therefore they have to be secure. Servers are usually well maintained, wire-connected and their resources are not usually constrained at all.

### 3.3. Assumptions

We assume that servers are secure and reliable. The number of wireless nodes is assumed to be huge compared to the number of servers. Due to being physically accessible and resource-constrained, wireless nodes are considered to be vulnerable. We assume an intruder can seize control of any wireless node in a minimum time *t_a_*.

There is a working service location system in the network, and it is secure and reliable. This article will not address the problems of deployment and operation of this service. We assume that every node in the network knows how to reach any particular service.

As redundancy is good for detecting and isolating attacks, any device providing useful information should be welcomed. Therefore, we assume that new wireless nodes can be added dynamically to our network without any restriction. Our architecture should assure that a continuous addition of bad nodes will not affect to the global behavior.

### 3.4. System Architecture

Our approach to the previously described threats is based on leveraging the two weapons that we have to detect and resist to attacks and failures: redundancy (spatial and temporal), and continuous adaptation. Also, we know that individual wireless nodes are vulnerable to attacks, and therefore no important decision should be made by a single node and no significant information should be stored in a single node.

We propose a software architecture based on many independent agents with simple and clear responsibilities. The term agent is heavily overloaded and should be defined more precisely. An agent in our system is an independent piece of software that is able to act on your behalf while you are doing other things (they are proactive), and it does this based on its knowledge of your preferences and the context. This knowledge is stored in servers and it is available to the network nodes through the use of passive services.

**Figure 2.**
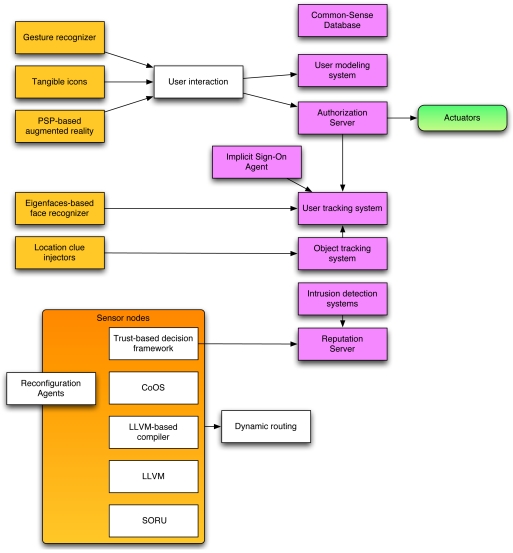
Main components in the security framework.

Figure 2 shows the main components of our framework. As can be seen, there is no direct communication between sensors and actuators, in order to avoid an intruder to modify the state of the environment while not preventing the free addition of sensor nodes to the system.

Individual sensor nodes are not trusted by default, and therefore the notion of trust is built dynamically by comparing a sensor with its neighbourhood. For this reason, every agent that needs to take into account data coming from sensor nodes or any derived information uses a trust-based decision framework that is further described below.

#### Sensor agents

Sensor agents are the simplest ones. They usually run on wireless nodes and provide measured data of external variables to the network, by sending messages to their routing agents. The message rate depends on the variation rate of the variable being monitored. This message rate should be enough to ensure that data items do not change too fast and therefore temporal redundancy can be used to detect failures or attacks.

Each sensor agent is associated to a sensor device and generates a sequence of measurements:

$$d_{\upsilon i}(t),d_{\upsilon i}(t+1),\ldots$$

where v is the variable being measured and i is the sensor agent id. Each data item is annotated with a time stamp, to detect temporal anomalies.

As previously stated, there is not a single routing agent for each sensor agent, and this agent decides randomly what routing agent to use for every message.

Although they do not consume data from other sensors, they need to maintain a trust table for their routing elements, that will only evolve with reputation information coming from the servers. Unlike in routing elements, the initial trust value for a routing element is positive, and the distribution of messages is uniform between all the routing nodes with positive trust.

#### Actuator agents

Actuator agents (light switches, electronic equipment controls, alarms, etc.) operate physically on the environment. They are especially critical because: 1) they are usually not redundant, and 2) any operation on them causes a physical effect on the environment. Therefore the nodes running actuator agents should be at least as tamper-resistant as the physical element they control. To ensure that an intruder cannot operate remotely on an actuator, only servers can send operation requests to these agents and they should use robust asymmetric encryption algorithms. As security and processing requirements are higher, these nodes are usually main powered.

The data flow goes from sensors to servers and from servers to actuators. There is no feedback from actuators to servers. So if an actuator is attacked, the assailant will not be able to access the other entities in the network.

Logically, an actuator works as a passive service, but it also develops a trust model of its environment, which is fed to the servers.

#### Aggregation agents

Aggregation agents reduce the redundancy by combining several data items using a known aggregation function. The only reason to apply these aggregations is to reduce the amount of data sent to the servers, allowing the system to scale. Trust computation implies also an aggregation of spatial and temporal redundant data that is held in a node.

#### Services

Services are passive elements that can be used by other nodes in the network. They usually run in servers. Some of the services that have important roles for security reasons are: object tracking system, user tracking system, user modeling system, and common sense database.

### 3.5. Trust-based Decision Framework

We follow the definitions and beliefs of Boukerch *et al.* in [26] concerning the distinction between trust and reputation. Trust is the degree of belief about the future behavior of other entities. Trust is subjective and it is based on past experiences. Reputation, on the other hand, is the global perception of an entity's behavior, and it is based on the trust that others hold on that entity. It is mostly objective and it has some influence in the evolution of trust in every node.

To consider a data item to be valid we use two consistency tests. The data item is said to be s-consistent or consistent with the spatial redundancy if it is consistent with the data provided by the majority of sensors that provide measurements of the same variable. For example, for a presence event from a PIR detector to be valid, the majority of nodes monitoring the same area should also detect presence. In this evaluation every sensor is weighted with the trust value the receiving node has about the source node.

A second way to discard bad data is to evaluate each data item against temporal data redundancy. Each routing element stores a limited set of previous values for each variable directly routed through itself. The data item is said to be t-consistent if the variation against previous data is normal for that variable. For example, if a temperature value changes drastically and it is not maintained during some time, maybe a routing element has been attacked.

Both properties, s-consistency and t-consistency, are dependent on the variable being measured. To model trust and reputation in our agent system, every node in the network maintains a trust table with entries for every relevant neighbor node. When a new node is discovered, the initial trust value is 0. Whenever a new message containing a new measurement of the external variable v arrives, trust on node i is recalculated as follows:

$$d_{\upsilon }(t)=A_{\upsilon }(\{\tau_{i}(t-1),d_{\upsilon i}(t)\})$$

$$\tau_{t}(t)=T(\tau_{i}(t-1),d_{\upsilon }(t),\bar{H_{\upsilon_{i}}})$$

where *d_v_*(*t*) is the value of the variable *v* built from neighbor measures and the previous trust value (*τ_i_*(*t* - 1)). *τ_i_*(*t*) symbolize the trust value of the evaluating node on node *i*. $\bar{H_{\upsilon_{i}}}$ represents all the data values of the variable *v* provided by node *i* that are stored in this node (history is usually truncated to reduce memory requirements). *A_v_* is an aggregation function that depends on the variable being measured, and it does not take into account data coming from a node with negative or zero trust value. *T* is also an aggregation function with these properties:

- If *τ_i_*(*t* - 1) is negative, the data item is discarded and no further processing is done for this message (repeated inconsistencies may lead to negative values of trust).
- If the new data element *d_vi_*(*t*) is s-inconsistent and t-inconsistent, it is stored in the local history (discarding the oldest value), but it is not taken into account for trust recalculation.
- If it is s-inconsistent with other sensors' data but t-consistent with previous values of the same sensor, trust on sensor i decreases.
- If it is s-consistent and t-consistent and current trust is positive, trust increases.

As can be seen, trust computation condenses historical information, and therefore it is bad, as we lose redundancy. On the other hand, resources are tightly constrained and we have to reduce storage requirements to a minimum.

To avoid some attacks, temporal disappearance means loss of positive trust (not negative). Whenever it appears again, it will get a 0 trust value. There is a second method to feed trust values back from redundancy analysis: reputation messages from the servers.

From time to time, nodes communicate their trust tables to the servers. This is done at the routing level by adding this trust information to messages that are being sent to the same destination. Servers are not resource constrained by assumption, and therefore they can store all the historical information for future analysis. The adequate combination of all the trust data of a zone generates the global reputation data:

$$\rho_{\upsilon i}(t)=R(\rho_{\upsilon i}(t-1),H_{\upsilon i})$$

where *H_vi_* represents all the history of data values of the variable *v* provided by sensor *i*, and *R* is another aggregation function. Well-behaved nodes increase their reputation; the reputation of bad-behaved ones decreases. Multiple agents can be running on the trust servers to look for attack evidences in the message history, and proactively reduce reputation values of suspect nodes.

Whenever a server decides that it has to act in the environment by modifying trust values for ill-behaved nodes, it broadcasts the reputation information of all the nodes in that zone. This message is repeated from time to time until the data the server receives from that zone is consistent with the global reputation information.

A wireless node will never take into account this reputation information unless it has been received from different routers (cluster heads). Thus, redundancy in routing paths and trust merging in secure servers allows us to feed good and bad reputation back to the network without being vulnerable to bad mouthing attacks.

The trust data sent to the servers is enough to detect most, if not all, common attacks. However, it is not enough to find the concrete faulty or compromised node, and therefore the servers would not be able to confine the attack. The solution we propose is to include the routing path in some of the messages. This way, by analyzing the paths of messages with t-consistent and s-consistent data it is easy to discard well-behaved nodes. Note that routing paths coming from a compromised node could have been faked. The confinement agents act directly by decreasing the reputation values of the suspect nodes.

A number of parameters (see Table 1) can be dynamically adjusted in order to adapt the environment to possible attacks. If the risk increases, we increase the local amount of redundancy around the affected area.

### 3.6. Routing Protocol

In order to improve network scalability and throughput, we use a clustering technique based on Random Competition based Clustering (RCC) [27] to construct a multi-level network structures. Previous approaches [28-30] group nodes into clusters, and within each cluster a node is elected as a cluster head. Cluster heads together form a higher-level network, upon which clustering can again be applied. This structure simplifies communication and makes it possible to restrict bandwidth-consuming network attacks like flooding to a single cluster.

For a wireless network with *n* nodes capable of transmitting at *Wbits/s*, according to [31], the throughput, *T*, for each node under optimal conditions is:

$$T=\Theta \left(\frac{W}{\sqrt{n}}\right)$$

Thanks to the clustering approach, in a two-level mobile backbone network where the number of nodes is *n* and the number of clusters is *m*, the throughput in the lower level becomes:

$$T_{1}=\Theta \left(\frac{W_{1}}{\sqrt{n/m}}\right)$$

and in the higher level:

$$T_{2}=\Theta \left(\frac{W_{2}}{\sqrt{m}}\right)$$

Node clustering, however, reduces redundancy and introduces single points of failure, as an intruder could control a whole zone by attacking its cluster head. The solution we propose is to introduce redundancy again. Every node in the network will have several cluster heads and will distribute messages randomly between them. This additional redundancy does not reduce the maximum throughput because at any given time the network structure is exactly the same as in the pure RCC scheme.

Of course, no node will ever select an untrusted cluster head. On the other hand, the s-consistency check required by the mechanism of reputation sharing would not be feasible without the non-determinism in the message paths introduced by the routing protocol. Therefore, the trust framework and the routing protocol cooperate in order to minimize the threats.

It may be argued that for every node to have two cluster heads, we need to double the backbone nodes so that there are twice as much backbone nodes in the coverage area. While it is true that more nodes have to belong to the backbone, this does not imply any reduction of the attainable throughput, as at any given time half the backbone nodes will not be used as such, and therefore the network structure remains exactly the same as in the pure RCC case. On the contrary, the burden of routing backbone messages is more distributed and therefore the penalty in energy consumption of being a cluster head is significantly reduced.

## 4. Attack Resistance

Nodes of a sensor network need to access, store, manipulate and communicate information. In AmI, nodes make decisions based on received data. Therefore, the system must guarantee data reliability. Some applications will require the use of sensitive information. In that case, measures to ensure data confidentiality should be taken into account. In this section, we will analyze the different kinds of attack that a sensor network is exposed to. The next sections classify the different threats attending to their primary focus.

### 4.1. Confidentiality Attacks

Confidentiality attacks attempt to access to the information stored in the sensor network. They can be further classified attending to the target of the attack:

- Attacks on the confidentiality of communications.
- Attacks on the confidentiality of node information (data generated in the sensor waiting to be sent to a server, service information stored in the network, and server information).

In a closed system with high-resources devices, information can be protected using cipher algorithm and physical access control. However, sensor networks are more vulnerable due to their characteristics:

1. Nodes have very limited resources.
2. Potential intruders may physically access to them.
3. Wireless communications.

The network can use well-suited cipher algorithms [32] to provide security against attacks to communications. Due to conditions 1 and 2, nodes are more vulnerable to the attacks than communications. Some approaches suggest ciphering stored data [33]. Nevertheless, a combination of logical (cryptography weakness and Trojan horses), and physical (DPA, SPA, micro-probing, reverse engineering) attacks could break the ciphering and access the information.

Due to the characteristics of the sensor nodes, it is not possible to secure its data against attacks. Even if we cipher the information in the devices, an attacker could use an approach based on logical and physical attacks that could break the ciphering. Since attackers have physical access to the nodes and nodes have limited resources, confidentiality should be based in the main characteristics of sensor networks: distribution and redundancy.

#### Attack on the confidentiality of node information

##### Sensor agents

In this kind of attack, the intruder accesses to the information stored in a sensor. If the attack succeeds, the attacker will obtain the information stored in it, but it is only raw data, not significant by itself. In addition, mapping that information with a concrete user is impossible because mapping information is stored in servers or distributed among a very large number of nodes. While the number of nodes holding some particular information remains much higher than the number of attacked nodes, attackers will not be able to obtain meaningful information.

##### Actuator agents

These agents do not store other information than the status of the physical device they control and the trust table for its routers.

##### Aggregation agents

By attacking an aggregation agent or a node that runs an aggregation agent, an intruder may gain access to redundant local raw data, but anything else. Redundant data is useful to discard bad data, but it gives no extra information.

##### Decision-making agents

They run in servers, which are not physically accessible, and have enough resources to keep the information secure.

#### Attack on the confidentiality of communications

In this attack, an intruder listens to the channel trying to obtain some information. Due to sensor redundancy and information distribution, the attacker should break all communications between sensors and routers to obtain some significant information. The use of some ciphering algorithms will help protecting the system. Since the network is big enough, an attacker that listens to the channel will obtain only a set of d*_v_*_i_(*t*). By definition, that set will not represent any meaningful information, so the attack will fail.

### 4.2. Denial of Service Attacks

A Denial of Service (DoS) attack is an attempt to interrupt, disrupt, or destroy services and operations in a system, which includes:

- *Jamming, collision and flooding*: These attacks consist in interfering in communication by sending messages through several protocol layers. The immediate effect of these attacks is the loss of part of the messages from the nodes of the affected area. The affected area depends on the layer in which it occurs. The upper the attack occurs on the protocol stack, the more it spreads. So the scope of these attacks could be zone or global depending on their dimension and the layer where they occur (physical, link, or transport layers). Wood and Stankovic [34] explain several countermeasures for these attacks: they suggest confinement, small frames, error-correcting codes and client puzzles.
- *Neglect and greed*: This simple form of DoS attack focus on a router vulnerability by arbitrarily ignoring all or some messages. It is especially dangerous in environments using hierarchical routes and static routing protocols. A possible solution would be a routing protocol with several paths available [34].
- *Misdirection, blackholes and wormholes* [35]: These attacks are very difficult to avoid, detect and confine. Authorization and monitoring have been proposed to avoid them. However, it is not possible to deploy a secure wireless sensor networks based exclusively on ciphering and authorization. It is necessary to supply additional techniques to reinforce the system. We will use redundancy again to detect these attacks. There exists some countermeasures consisting on enhanced protocols [36], however they require too many resources to be used in tiny nodes.

Now, we will show how our system can detect and confine the denial of service attacks.

#### Jamming, collision and flooding

Whether it is jamming, collision or flooding, the effects in the network are similar: loss of messages and node disappearance. The seriousness and extension of the attack depends on the number of nodes, the stack layer where it takes place and several other parameters. Nevertheless, it leads some nodes to disappear. As no new value from these nodes arrives to the routers, as trust tables are sent to the servers, the global trust service will soon discover that the latest values coming from these nodes are obsolete and it will mark them as lost.

The detection of the attack can be performed when a group of nodes in the same area disappears suddenly. If a node with positive reputation disappears temporally its reputation will be decreased. This measure will also affect directly to the routers in the area. Therefore, a message will not be sent through an affected router, avoiding the zone.

Flooding attacks could be more dangerous if messages are scattered and the whole network is affected. But if the reputation of a faked node is decreased, its forwarded messages will not be routed and, therefore, harm will not spread.

#### Neglect and greed, and blackholes

A router may neglect to route all or some messages, but every node has two or more routers that are used randomly, and so eventually the messages will arrive to the destination.

Some of the messages include their own route, and the servers analyze the routes of consistent messages to find out the routers which do not route properly. A feedback of negative reputation for these routers will cause messages to follow other routes avoiding these malicious routers.

#### Misdirection and wormholes

Local attacks can get worse if the compromised node stops routing properly, changes the values notified by some sensors, or teleports messages to other area of the network.

A combined use of localization information (object tracking system), and route analysis for messages coming from the same area (redundancy in routing elements will ensure that not every message will go through the wormhole), allows to discover easily the bad routers. There are some proposals similar to this one, like in [37, 38] where the authors propose a method based on location information of each node join to identity information in messages or like in [39] where a statistical process of network data is used to detect wormholes. Our system manages the required data so both are feasible solutions.

Again, once the malicious routers have been detected, it is possible to confine the compromised nodes by decreasing their reputation. If a router has a low reputation it will be probably not chosen for routing messages. And redundancy in routing elements ensures that the new reputation table will eventually arrive to any node in the network.

Trust tables going from the sensor nodes to the servers and reputation tables coming back from the servers can also be altered by a compromised node, but redundancy again allows discarding bad messages.

### 4.3. Integrity Attacks

Integrity attacks try to alter the normal behavior of the system by modifying the data stored in nodes. Although DoS attacks can be considered as integrity attacks as service interruption is one kind of bad behavior, we prefer to treat them separately because here the focus is on the data, instead of the communications.

#### Tampering and homing

These attacks are very difficult to avoid due to the weakness of wireless nodes. But these are clear cases of local attacks. Local or node attacks are not relevant for our network model, since redundancy allows losing nodes without any impact in the behavior. Negative reputation can be used from the servers in order to confine these attacks. Even if integrity of individual nodes is difficult to achieve, the use of redundancy can reduce or eliminate the impact on the global system.

### 4.4. Identity Attacks

Malicious nodes can pretend to be other nodes in order to implement one of the attacks mentioned above. We will consider four different types: clone, thief, mole and sybil.

- The *clone attack* consists in duplicating an operating node. Both nodes, simultaneously, communicate with the same identity.
- In the *thief attack*, a malicious node steals an operating node its identity and replaces it in the network. The malicious node stops original node's operation and takes advantage of its reputation and trust levels.
- A *mole* is a malicious node that behaves as a well-operating node, with a fabricated identification, to achieve high levels of trust and reputation. Once inside, it can attack the system from a privileged position. A variation is the *on-off* attack, where the malicious node behaves well and badly alternatively, in order to maintain a high average level of trust.
- The *sybil attack* occurs when a malicious device presents multiple identities, as if it were multiple nodes, in order to control a substantial fraction of the system. This attack reduces the effect of the system's redundancy without the need of numerous physical nodes. The attacks can be performed at any layer of the protocol stack, but they are more profitable in the upper layers, like network or application.

The first three attacks are carried out by individual malicious nodes, and they can be considered special cases of the sybil attack. The sybil attack was first introduced in [24]. Newsome [25], Karlof [40] and Zhang [41] make thorough descriptions of the taxonomy, threats and countermeasures of identity attacks, focusing on the sybil attack. We can find three main types of solutions to the identity attacks: resource testing, cryptography and location-based.

Resource testing solutions assume that devices are limited in some resource [24]. The solutions consist in testing a limited resource and checking that each identity has no less capability than a physical node. The resource tested in wireless sensor networks, according to Newsome [25], is the radio communication capability, considering that a device can access only to one radio channel at a time. Each identity has a channel assigned and they must send a message through it simultaneously. The system detects an identity of a sybil attack when it receives no message in its channel. Accurate synchronization between the monitoring devices is needed and, if we have more identities than channels, we can't perform the test to every identity at the same time, so the detection rate decreases.

Cryptography schemes base their efficiency in secure communications, and the different solutions differ in how to establish the keys: the key agreement process. They can have a key server with the public key of all nodes, and only establish a key through the key server. Another scheme uses the self-enforcing scheme approach, based on asymmetric cryptography with public key. Efficient implementations of Elliptic Curve Cryptography (ECC) Cipher Suites can be used in sensor networks to establish secure links, but it is not enough to avoid the sybil attack, because a malicious device may have more resources than the normal nodes. The third key agreement mechanism is key pre-distribution scheme [42–44]. In these systems each sensor has a subset of the system keys and a secure link is established between nodes which have at least one key in common. If a node is compromised, several keys are known by the malicious device. If more nodes are compromised, the attackers can obtain a substantial fraction of the system keys.

Location based solutions [45, 46], check that no identities are at the same position. The solutions assume that the sensor nodes are static, but real AmI applications have heterogeneous networks, with static and moving nodes. The accuracy of the location system should be high due to the high density of sensors inherent to AmI applications.

Clone, thief and mole attacks use only one identity, so their effect is the same as compromising a sole node. It is proved, as shown in previous sections, that the system adapts to individual attacks. If the node's behavior is consistent with the other nodes, the attack is undetectable, but the information obtained is not significant. In the clone attack the system can detect that the same identity is being used in two different locations, so the server would reduce the reputation of both nodes.

On the other hand, the sybil attack can be dangerous to the system because it reduces the effect of the system's redundancy. Our architecture solves the sybil attack problem by reducing its attack rate. When an aggregation agent receives information from an unknown node, the trust level default value is zero. This is enough to send data from this node to the servers to collect behavior history, but not enough to be taken into account in any decision or aggregation. If the node behaves correctly, its reputation will grow eventually, but always at a controlled rate. If many sensors are appearing in a short time in the same area, the required time to have positive reputation will increase.

## 5. Empirical Results

The proposed architecture has been simulated extensively to evaluate its behaviour in presence of attacks of very different nature.

The most common attacks are detected and confined immediately with no other effect in the surroundings. Ill-behaved nodes will never get high reputation, but even for mole attacks, an attacker would need to add at least as many nodes as there are in the attacked area in order to have any influence in the decision. But even in that case, it would be easily detected by software agents analyzing the servers data. In our case, we use self-organizing maps (SOM) and genetic algorithms to detect anomalies in the system behavior. These agents can immediately confine the attack by changing the global reputation of the misbehaving nodes and correcting the affected neighbours'. As routes are non-deterministic, attacks to routing elements only delay the response time of the system by 1/*N_r_*.

**Figure 3.**
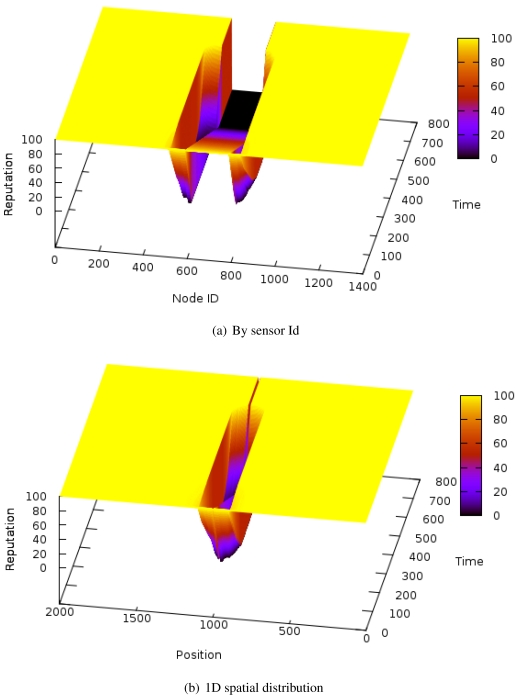
Reputation evolution for a sybil attack.

One of the most significant results is the behavior of the system when a compromised node tries to impersonate many existing sensors (a sybil attack). Figure 3(a) shows the evolution of the reputation of every node when the sybil attack is introduced. The Y-axis represents time, while the X-axis represent the node identifiers, ordered by their projection in one dimension but not preserving distance proportions. Figure 3(b) is similar but preserving distance proportions between nodes (note that the compromised node is just one point in the X-axis with many associated identifiers). In this last case, the reputation of the nodes whose identity is being stolen would decrease at the same rate as the sybil identities, but they have been omitted from the graph for clarity purposes. The sybil attack uses 200 different identifiers out of 1400 total nodes in the system. The reputation server is located at the origin. This is an extreme case to better show the effects in the attack neighborhood.

**Figure 4.**
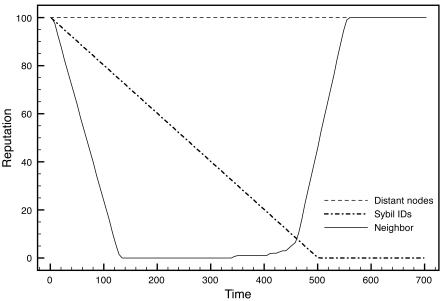
Reputation evolution for three different nodes during a sybil attack (the attacked node, a neighbor, and a distant one).

It is noteworthy that our algorithm allows very fast confinement of the attack, by reducing immediately the reputation of the neighbor elements. Figure 4 shows the evolution of the reputation of three different kinds of node identities during a sybil attack: the identities used by the attacked nodes, the identities of neighbor nodes, and identities from distant nodes. The system can not distinguish between the real IDs and the faked IDs, and therefore both are finally discarded from the reputation system. As far as the number of attacked nodes remain low compared to well-behaved nodes, this should not be a problem. As soon as the affected nodes are ignored (when they finally end with null reputation), the neighbor elements recover their initial reputation. Also, routing path analysis avoids significant reductions of the real nodes being impersonated.

In our system, trust information is not shared directly between the sensor nodes, it is sent to the reputation server. Therefore, our trust framework is not vulnerable to attacks based on an inconsistent behavior in the time domain (on-off attacks, clone attacks, and thief attacks), or the user domain (conflicting behavior attack). The attacked node can not influence directly on its neighbors unless there is a majority of badly-behaved nodes with high trust levels.

The node reputation is the only trust-related information that is shared by the network. This reputation is elaborated by the reputation server, which has more information than any individual node, and also has more resources to avoid local attacks. Attacks to the reputation messages, or even the generation of these messages, is avoided by the multiple routing paths, and because no information is trusted unless it is t-consistent and s-consistent.

## 6. Conclusions

Wireless Sensor Networks are based on many wireless, low-cost, low-power, and low-resources nodes. These characteristics and the possibility to access physically to the nodes make them highly vulnerable to attacks. Cryptography appears as clearly insufficient to maintain data confidentiality and integrity in the network.

We have proposed a holistic solution that assumes this node vulnerability to address security issues in an intelligent ambient based on massive wireless sensor networks.

Redundancy and fast continuous adaptation have been identified as the key weapons to defend the system against attacks, and they are used consistently to cope with security issues at different levels.

The proposed architecture is based on an agent system with supporting services. Data flows from the sensors to the servers, where it is processed returning relevant semantic enhancements back to the environment. Agents running in insecure wireless nodes never hold a significant information unit, what preserves global confidentiality, and decisions are made in servers, what preserves integrity if redundancy is used adequately.

Most attacks are detected by the analysis of the redundant data available locally in every routing element and globally collected in the servers. Decisions at different levels are supported by a trust-based framework where trust data only flows from the sensors to the servers and reputation only from the servers to the sensors. Non-deterministic routes allows to detect and confine misbehaving routers.

The resulting approach takes into account practical issues, such as resource limitation, bandwidth optimization, and scalability. Based on these results we claim that our approach provides a practical solution for developing secure applications on top of wireless sensor networks.

## Figures and Tables

**Table 1.** Parameters that can be adjusted dynamically to adapt the environment to possible attacks.

Parameter	Description
Redundancy-related	

*N_p_*	Number of reputation tables stored in a node.
*N_d_*	Number of values stored for each sensor/value pair.
*N_r_*	Number of routers per node.

Adaptation-related	

*t_τ_*	Time between trust data messages sent to the reputation servers.
*t_ρ_*	Time between reputation data messages from the servers to the nodes.
*t_v_*	Time between sensor data messages from the sensor nodes to the network.
*t_r_*	Minimum time between messages containing route information.
